# Semaphorin 3A, a potential immune regulator in Familial Mediterranean Fever

**DOI:** 10.1186/1546-0096-13-S1-O46

**Published:** 2015-09-28

**Authors:** D Rimar, I Rosner, G Slobodin, N Boulman, L Kaly, M Rozenbaum, Z Vadasz

**Affiliations:** 1 Bnai-Zion medical center, Rheumatology, Haifa, Israel

## Introduction

Semaphorin 3A (sema3A) plays a regulatory role in immune responses, mainly affecting the activation of regulatory T cells. It has been found to correlate with disease activity in rheumatoid arthritis and systemic lupus erythematosus. Familial Mediterrenean Fevere (FMF) is an autoinflammatory disease; yet a possible role for regulatory T cells has been described.

## Aim

To evaluate the expression of sema3A in peripheral blood, on B cells and on regulatory T cells, of FMF patients during attack, in remission and with smoldering disease, in comparison with healthy controls.

## Methods

17 FMF patients in attack and in remission, 8 FMF patients with smoldering disease and 12 healthy controls were enrolled. Smoldering disease was defined in FMF patients with high CRP level (above 20mg/dL) without concurrent symptoms of serositis, arthritis nor concomitant known infectious disease. Sema3A in peripheral blood was measured by ELISA and expression of sema3A on regulatory T cells and regulatory B cells was evaluated by FACS analysis. FMF patients were evaluated for demographics and disease severity by the Mor severity score.

## Results

The 3 groups of patients: a. FMF in attack and remission, b. FMF with smoldering disease and c. healthy controls were similar with regard to age (38.1±10.2 vs.48±15 vs.43.1±3.4) and female gender (8 [47%] vs. 4 [50%] vs.5 [41%]). The age of onset was 10 years ±7.9 in group a and 15 years ±6.3 in group b. The mean colchicine dose was similar between groups a and b (1.9±0.48 vs. 2.25±0.5) and so was the Mor severity score (3.2± 1.9 and 2.8± 1.6).

Semaphorin 3A expression on regulatory T cells in FMF patients during an attack was lower than in remission and in healthy controls (57.2%± 8.3 vs. 77.2%±10.3 vs. 88.7%± 3.6%, p<0.01) but similar to patients with smoldering disease.

Semaphorin 3A expression on regulatory B cells were lower in FMF patients during an attack than FMF in remission and in healthy controls (72.9%±8.5 vs. 83.4%±5.8 vs. 82.6%±6.4 ng/ml, p<0.05), but was similar to patients with smoldering disease.

Semaphorin 3A concentration in peripheral blood as evaluated by ELISA was lower in FMF patients during an attack, in smoldering disease and in remission than in healthy controls (242.3±9.8 ng/ml vs. 258.9±11.5 ng/ml vs. 232.5±22.7 ng/ml vs. 323.3±160.2 ng/ml, p<0.05).

## Conclusion

Sema3A expression on regulatory T cells and regulatory B cells is low in FMF patients during an attack and in smoldering disease compared to the expression in FMF remission and in healthy controls. Regulatory T cells have been described to increase one week after an attack and were speculated to have a role in the termination of FMF attacks. A decrease in sema3A in an attack and normalization at remission may help explain the decrease and subsequent normalization of regulatory T cells. The role of regulatory T cells and semaphorin 3A in termination of FMF attacks needs further evaluation on a wider scale.

